# Confirmation of a non-synonymous SNP in *PNPLA8* as a candidate causal mutation for Weaver syndrome in Brown Swiss cattle

**DOI:** 10.1186/s12711-016-0201-5

**Published:** 2016-03-18

**Authors:** Elisabeth Kunz, Sophie Rothammer, Hubert Pausch, Hermann Schwarzenbacher, Franz R. Seefried, Kaspar Matiasek, Doris Seichter, Ingolf Russ, Ruedi Fries, Ivica Medugorac

**Affiliations:** Chair of Animal Genetics and Husbandry, Ludwig-Maximilians-Universitaet Muenchen, Veterinaerstr. 13, 80539 Munich, Germany; Chair of Animal Breeding, Technische Universitaet Muenchen, Liesel-Beckmann-Straße (Hochfeldweg) 1, 85354 Freising-Weihenstephan, Germany; ZuchtData EDV-Dienstleistungen GmbH, Dresdner Straße 89/19, 1200 Vienna, Austria; Qualitas AG, Chamerstr. 56, 6300 Zug, Switzerland; Institute of Veterinary Pathology, Ludwig-Maximilians-Universitaet Muenchen, Veterinaerstr. 13, 80539 Munich, Germany; Tierzuchtforschung e.V. Muenchen, Senator-Gerauer-Str. 23, 85586 Poing, Germany

## Abstract

**Background:**

Bovine progressive degenerative myeloencephalopathy (Weaver syndrome) is a neurodegenerative disorder in Brown Swiss cattle that is characterized by progressive hind leg weakness and ataxia, while sensorium and spinal reflexes remain unaffected. Although the causal mutation has not been identified yet, an indirect genetic test based on six microsatellite markers and consequent exclusion of Weaver carriers from breeding have led to the complete absence of new cases for over two decades. Evaluation of disease status by imputation of 41 diagnostic single nucleotide polymorphisms (SNPs) and a common haplotype published in 2013 identified several suspected carriers in the current breeding population, which suggests a higher frequency of the Weaver allele than anticipated. In order to prevent the reemergence of the disease, this study aimed at mapping the gene that underlies Weaver syndrome and thus at providing the basis for direct genetic testing and monitoring of today’s Braunvieh/Brown Swiss herds.

**Results:**

Combined linkage/linkage disequilibrium mapping on *Bos taurus* chromosome (BTA) 4 based on Illumina Bovine SNP50 genotypes of 43 Weaver-affected, 31 Weaver carrier and 86 Weaver-free animals resulted in a maximum likelihood ratio test statistic value at position 49,812,384 bp. The confidence interval (0.853 Mb) determined by the 2-LOD drop-off method was contained within a 1.72-Mb segment of extended homozygosity. Exploitation of whole-genome sequence data from two official Weaver carriers and 1145 other bulls that were sequenced in Run4 of the 1000 bull genomes project showed that only a non-synonymous SNP (rs800397662) within the *PNPLA8* gene at position 49,878,773 bp was concordant with the Weaver carrier status. Targeted SNP genotyping confirmed this SNP as a candidate causal mutation for Weaver syndrome. Genotyping for the candidate causal mutation in a random sample of 2334 current Braunvieh animals suggested a frequency of the Weaver allele of 0.26 %.

**Conclusions:**

Through combined use of exhaustive sequencing data and SNP genotyping results, we were able to provide evidence that supports the non-synonymous mutation at position 49,878,773 bp as the most likely causal mutation for Weaver syndrome. Further studies are needed to uncover the exact mechanisms that underlie this syndrome.

**Electronic supplementary material:**

The online version of this article (doi:10.1186/s12711-016-0201-5) contains supplementary material, which is available to authorized users.

## Background

Since the first case report of a new autosomal recessive disorder in purebred Brown Swiss cattle was published in 1973 [1], efforts have been undertaken to identify the gene that causes bovine progressive degenerative myeloencephalopathy or the so-called Weaver syndrome. However, although the genetic mechanisms that underlie other heritable diseases in the Brown Swiss/Braunvieh breed [2–4] have been elucidated, the mutation that is responsible for Weaver syndrome remains unknown to date. The first symptoms of this disorder are observed at 5–8 months of age and include ataxia, hind leg weakness and a weaving gait [5, 6]. Normal sensorium and spinal reflexes are maintained throughout the course of the disease while affected animals progressively lose control over their hind quarters until they become recumbent and have to be euthanized for animal welfare reasons [5, 7]. The histopathological lesions are mainly observed in the white matter of the spinal cord and the highest concentration of lesions are observed in the cranial thoracic segment; the grey matter remains unaffected [8–10]. Degenerative changes comprise axonal swelling (spheroids), disintegration of myelin sheaths and myelin loss as well as axonal atrophy to the point of complete loss and status spongiosus [8, 9, 11]. Similar alterations are observed in the pyramids and the inferior olivary nuclei, whereas those in the cerebellum consist of a reduced number of Purkinje cells [9].

This neurodegenerative disease originated in the United States and was introduced into Braunvieh herds in Canada [12] and Europe [13] through the use of American Brown Swiss semen for artificial insemination (AI), with cases reported in Switzerland [10], Germany [5, 14], Italy [15] and Denmark [16] approximately 20 years after the import of the first carriers. In Germany, the occurrence of Weaver syndrome reached its peak between 1989 and 1991 with more than 20 cases documented by the Institute of Veterinary Pathology of the Ludwig-Maximilians-Universitaet (LMU) Muenchen in 1989 alone; the exact number is probably even larger because of unknown cases due to misdiagnoses and inconsistent reporting. Frequencies of the deleterious Weaver allele as high as 6.79 % in the Austrian [17] and 6.27 % in the American Brown Swiss population [18] were reported. In a first attempt to limit the occurrence of new Weaver cases, animals with two or more affected progeny for which parentage verification was done by blood testing were excluded from further breeding. A genetic test for detection of carriers became available in 1993 when Georges et al. [19] identified a close link between the Weaver locus and microsatellite TGLA116. In the following years, an improved indirect genetic test that included five additional microsatellite markers was developed [20]. Based on haplotypes of these six microsatellites that comprised two outer (RM188 and BM6458) and four core markers (MAF50, RM067, TGLA116 and BM1224), animals with an estimated risk of carrying the Weaver allele higher than 95 % were consequently excluded from breeding (Ivica Medugorac, Ingolf Russ, unpublished observations). The successful implementation of this strategy resulted in a steadily decreasing number of Weaver-affected animals and the last case was diagnosed by the LMU Institute of Veterinary Pathology in 1997. However, the Weaver allele has not been completely eliminated from the population and the estimated frequency ranges from 3.28 to 4.01 % in the Austrian [21] and is about 2.6 % in the American Brown Swiss population [22]. In 2013, McClure et al. [23] discussed the possibility of an even higher frequency due to possible recombination between the Weaver locus and the microsatellite markers of the diagnostic test, which may lead to false negative results for active breeding animals. Thus, the frequency of the deleterious Weaver allele may continue to increase without being noticed and the occurrence of new Weaver cases would only be a question of time. Although the fine-mapping approach that was used by McClure et al. [23] was not successful in detecting the causal mutation, the Weaver locus was narrowed down to a 5-Mb window between 48 and 53 Mb on BTA4 (BTA for *Bos taurus* chromosome), which led to the identification of 41 diagnostic single nucleotide polymorphisms (SNPs) and to the definition of a common haplotype associated with the Weaver phenotype. Indirect diagnosis by SNP-based imputation and haplotype analysis [23] suggested that in spite of the exclusion of high-risk breeding animals and complete absence of Weaver cases over the last two decades, some of the animals used in current breeding programs could be Weaver carriers. However, in some cases, in-depth pedigree analyses of such potential carriers did not identify any confirmed Weaver carrier ancestors, which can be explained by: (1) inaccurate or incomplete pedigree records, (2) the presence of a large number of previously undetected carriers in the current Braunvieh/Brown Swiss population, or (3) a relatively recent causal mutation, thus the frequency of the original haplotype containing the ancestral allele may still be high, leading to false positive imputation results.

The aim of this study was to identify the allele(s) that cause Weaver syndrome and thus to provide the basis for a direct gene diagnosis, effective monitoring of the genetically active Braunvieh/Brown Swiss population and also for future studies on the exact molecular genetic mechanisms that underlie this neurodegenerative disease.

## Methods

### Animal samples and phenotypes

In this study, we used a population that consisted of 34 Original Braunvieh individuals and a group of 126 purebred Brown Swiss and Brown Swiss × Braunvieh animals. Of these 160 animals, 86 were Weaver-free, 31 Weaver carriers and 43 Weaver-affected. Disease status was assigned based on neuropathological records from the LMU Institute of Veterinary Pathology that confirmed that the lesions and alterations were characteristic of the Weaver syndrome. Weaver carriers were identified by progeny-testing and required that an animal had to have at least two Weaver-affected offspring with proven ancestry to be declared an official carrier of the Weaver syndrome.

Since the last case of Weaver syndrome that was reported in 1997, no biological samples from live Weaver-affected animals were available. Instead, we used blood and DNA samples that were available from studies performed during the late 1980s and early 1990s for analyses. For some cases, additional samples of formalin-embedded spinal cord tissue and sections of the spinal cord that had been stored in the archives of the LMU Institute of Veterinary Pathology were investigated. Since all the samples used in the current study originated from previous routine tests for paternity and determining an animal’s Weaver status, ethical approval was not required.

### Genotypes

Genotyping was performed on 43 Weaver-affected, 28 Weaver carriers and 51 Weaver-free animals using the Illumina Bovine SNP50 BeadChip (Illumina, San Diego, USA). Data on 34 Original Braunvieh animals were also available from a previous research project [24]. Genotypes of four additional animals (three Weaver carriers and one Weaver-free animal) were provided by Braunvieh Schweiz (Zug, CH). The physical positions of all SNPs were determined according to the UMD 3.1 *Bos taurus* reference assembly [25]. SNP call rates for all animals were higher than 0.95, thus it was not necessary to exclude any animals because of low genotyping success.

For further analysis, SNPs were filtered based on the following exclusion criteria: (1) unsuccessful genotyping for more than 5 % of the animals, (2) frequent paternity conflicts for animals with known paternity, (3) unknown or ambiguous map positions in the reference genome, (4) SNPs with a level of heterozygosity <0.05 and (5) SNPs located on chromosomes other than BTA4 since the Weaver locus has been mapped to BTA4 by Georges et al. [19]. After filtering, 1958 SNPs remained for analyses.

### Linkage/linkage disequilibrium mapping on BTA4

In order to fine map the Weaver locus, a combined linkage disequilibrium and linkage (*cLDLA*) method equivalent to that proposed by Meuwissen et al. [26] was applied, which allows the use of data on both confirmed carriers and affected animals.

First, haplotype reconstruction and imputation of missing genotypes were conducted using the program *BEAGLE3.0.4* [27], which is based on a Hidden Markov model and exploits the linkage information contained in the relationships between genotyped animals in the pedigree. To increase the accuracy of haplotype reconstruction, 8153 additional animals that were otherwise not part of our analyses were included. The whole dataset consisted of 168 parent–offspring trios, 3212 parent–offspring pairs and 5134 unrelated animals. The 43 Weaver-affected animals in the population used for mapping were divided in two categories, i.e. 22 parent–offspring pairs and the remaining 21 animals for which no genotype information on the parents was available.

To avoid an inflation of false-positive results due to population stratification in the final mixed linear model, the genome-wide unified additive relationships (UAR) between all animals were estimated [28]. The principal components of the UAR-matrix were determined in *R* [29]. The R package *paran*, which is an implementation of Horn’s parallel analysis [30], was then used to identify the main principal components that explain more than 90 % of the genetic variance. The first 60 principal components were integrated in the mixed linear model.

To account for local haplotype relationships and, thus, linkage disequilibrium, sliding windows of 40 consecutive SNPs along BTA4 were used. Following the method described by Meuwissen and Goddard [31], the locus identity by descent (*LocIBD*) was estimated for each 40-SNP window midpoint, i.e. between SNPs 20 and 21. Subsequently, the procedure suggested by Lee and Van der Werf [32] for additive genetic relationship matrices (**G**_**RM**_) was applied to convert the resulting *LocIBD* matrices into diplotype relationship matrices (**D**_**RM**_).

Finally, a variance component analysis at the midpoint of each of the 40-SNP sliding windows was conducted using *ASReml* [33]. The mixed linear model was as follows:

$$ {\mathbf{y}} \, = \, {\mathbf{X}}{\varvec{\upbeta}} \, + \, {\mathbf{Zq}} \, + \, {\mathbf{e}}, $$

where **y** is a n × 1 vector of phenotypes of the investigated trait. In order to map Weaver syndrome as a quantitative trait, phenotypes were converted into numerical values as follows: 1.0 for Weaver-free animals, 2.0 for confirmed Weaver carriers and 3.0 for Weaver-affected animals confirmed by pathological records. In practice, Weaver syndrome was diagnosed based on clinical observations in a three-step process: (1) the first clinical observation and diagnosis was carried out by local veterinarians on the farm, (2) if the farmer agreed, clear-cut Weaver cases were transported to the Clinic for Ruminants (LMU, Oberschleißheim) for additional clinical observation, and (3) the Weaver cases that were confirmed in step (2) were euthanized and pathologically examined by the Institute of Veterinary Pathology (LMU, Munich). Some Weaver cases were only supported by observations from steps (1) and (2) because final pathological proof was not performed mainly because of either schedule difficulties, i.e. no capacities were available at the Institute of Veterinary Pathology on the desired date, or of premature death of Weaver animals identified in steps (1) and (2). For clinically-confirmed animals that lacked pathological records, we applied the following interpolated phenotype values: 2.75 for animals with a confirmed carrier ancestor on both the maternal and paternal side of the pedigree with a maximum interval of two generations between them; 2.5 for animals with an interval of three or more generations between themselves and one or both carrier ancestors; 2.25 for animals with only one official carrier in the pedigree; and 2.0 for animals with missing pedigree data. **β** is a vector of fixed effects that contains the overall mean *µ* and the 60 principal components. Random additive genetic effects based on the **D**_**RM**_ are included in **q**, a vector that follows a normal distribution, i.e. **q** ~ N(0, **D**_**RMp**_$\sigma_{q}^{2}$), where **D**_**RMp**_ is the diplotype relationship matrix at position *p* of the putative quantitative trait locus (QTL), while **e** is a vector of random residual effects with **e** ~ N(0, **I**$\sigma_{e}^{2}$), where **I** is an identity matrix. **X** and **Z** are incidence matrices relating to fixed and random QTL effects. Both vectors **q** and **e** are assumed to be uncorrelated and have a normal distribution with mean 0 and variance $\sigma_{q}^{2}$ and $\sigma_{e}^{2}$.

For each 40-SNP window, *ASReml* simultaneously estimates the maximum log-likelihood, variance components and the fixed and random effects by taking into consideration the IBD probabilities of a putative QTL at the window midpoint. To compare the resulting log-likelihoods of the model assuming a QTL (alternative hypothesis, H1) with a model excluding a QTL at this location (null hypothesis, H0), a likelihood ratio test statistic (*LRT*) was used. It was calculated as follows:

$$ LRT = \, - 2 \times \left( {\log \,{\text{likelihood}}\left( {{\text{H}}0} \right) - \log \,{\text{likelihood}}\left( {{\text{H}}1} \right)} \right), $$

and shows a Chi square distribution with one degree of freedom [34]. The SNP window for which the difference between both likelihoods reaches its maximum is most likely to harbor the QTL.

In order to maintain the rate of false discoveries (type I errors) at a low level, a rather conservative significance threshold was chosen, i.e. *LRT* = 25.223, which corresponds to a Bonferroni-corrected *P* value of less than 5.11 × 10^−7^ (0.001/1958 = 5.11 × 10^−7^). For peaks with a *LRT* higher than 25.223, confidence intervals (CI) were determined by the 2-LOD drop-off method (1 LOD (log of odds) = 4.605) [35, 36]. The identified region was then compared with a map of annotated genes to identify possible candidate genes.

### Homozygosity mapping and identification of a common haplotype on BTA4

In order to compare the *cLDLA* method with classical homozygosity mapping, the same Weaver-affected and Weaver-free animals that were included in the *cLDLA* approach were used in a case–control design following the *ASSHOM* procedure in Charlier et al. [37]. In a parallel approach, genotypes of a subset of 31 carrier animals (progeny-confirmed) and 13 Weaver-affected direct offspring (status confirmed by pathological records and with at least one genotyped parent) were manually analyzed, which led us to identify a common haplotype that was compared to the interval detected by homozygosity mapping.

### Exploiting whole-genome sequence data for the identification of candidate causal mutations

Sequence data from Run4 of the 1000 bull genomes project [38] were exploited to identify candidate causal mutations for Weaver syndrome in Braunvieh cattle. The Run4 data consist of full sequence information for 1147 animals that represent 29 breeds including 59 Braunvieh animals. Two known Weaver carriers (*TARGET*-BSWUSAM000000174360 and *MODERN*-BSWUSAM000000156458) are among the sequenced Braunvieh animals. All sequence variants (SNPs, short insertions and deletions) located within the 1.72-Mb segment (between 48,688,283 and 50,412,884 bp) of extended homozygosity were considered for the identification of candidate causal mutations. Those mutations were filtered for variants for which the two known Weaver carriers were heterozygous, while all other animals were homozygous for the reference allele.

### Targeted SNP genotyping by PCR-RFLP

The candidate SNP (rs800397662) on BTA4 at position 49,878,773 bp was analyzed by PCR-RFLP on 103 animals (40 Weaver-affected and 63 Weaver carriers) for which DNA samples (that had been prepared in the late 1980s and early 1990s) were still available. The DNA segment containing the candidate mutation was amplified by PCR using the following primers *5′*-*CAAAGGCTTTTGGCGTTATC*-*3′* and *5′*-*GCAAACAGAAGCAGATCCTTTT*-*3′* and subsequently treated with the site-specific restriction enzyme *RsaI*. Fragments were separated according to size and visualized by 2 % ethidium bromide-stained agarose gel electrophoresis.

For the second variant (rs442854880) that was associated with the Weaver status based on whole-genome sequence data but was located outside the segment of extended homozygosity at Chr4:50,858,538 bp, the same procedure was applied on samples of 16 Weaver-affected animals and 30 confirmed carriers (restriction enzyme *RsaI*, primers *5*-*GATCGAGCAGCTGAAAAAGG*-*3′* and *5′*- *AAGTCACCATGGGAAACCTG*-*3′*).

### Pathological re-evaluation of animals with conflicting PCR-RFLP results

For animals that showed discrepancies between the anticipated disease status and the genotyping results, histopathological sections of the spinal cord which had been kept in the archive of the LMU Institute of Veterinary Pathology were re-evaluated. If embedded spinal cord tissue was also available, additional hematoxylin–eosin-stained sections were prepared. These sections were then evaluated by an experienced veterinary neuropathologist in the light of the latest scientific knowledge.

## Results

### Linkage/linkage disequilibrium mapping on BTA4

**Fig. 1:**
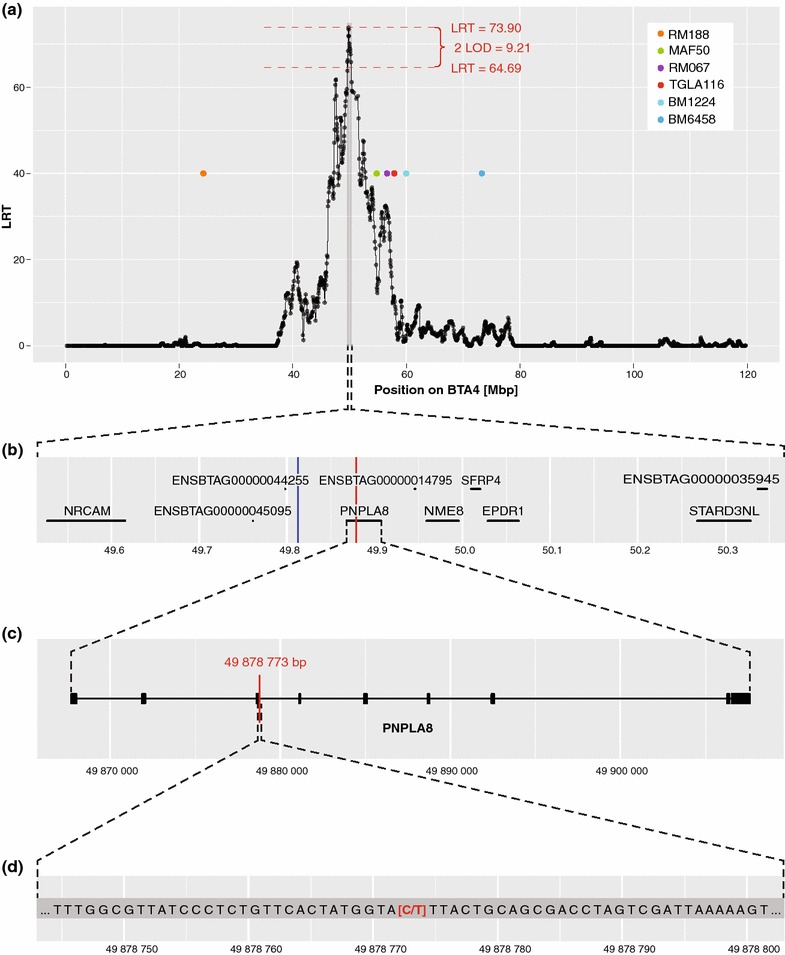
Identification of a candidate causal mutation at position 49,878,773 bp on BTA4. **a** Results of the combined linkage/linkage disequilibrium mapping approach. *Colored dots* indicate the positions of the six microsatellite markers (RM188, MAF50, RM067, TGLA116, BM1224, BM6458) of the indirect genetic test for Weaver syndrome. A maximum peak value (*LRT* = 73.9) was detected between microsatellite markers RM188 and MAF50 at position 49,812,384 bp. The 2-LOD drop-off method used to determine the corresponding confidence interval sets its boundaries at positions 49,514,652 and 50,367,484 bp. **b** Detailed overview of the confidence interval between 49,514,652 and 50,367,484 bp. Six genes (*NRCAM*, *PNPLA8*, *NME8*, *SFRP4*, *EPDR1*, *STARD3NL*), two novel microRNA genes (*ENSBTAG00000045095*, *ENSBTAG00000044255*) and two uncharacterized protein coding genes (*ENSBTAG00000014795* and *ENSBTAG00000035945*) are located within this confidence interval. The *vertical blue line* indicates the position of the maximum peak value (*LRT* = 73.9, 49,812,384 bp). The *vertical red line* indicates the position of the candidate causal mutation at position 49,878,773 bp (rs800397662), which was identified by analyzing whole-genome sequence data. **c** Detailed structure of the *PNPLA8* gene that carries the candidate causal mutation. *Bold sections* represent exons, thin sections represent introns. The *vertical red line* indicates the position of the candidate causal mutation. **d** The 41-bp DNA sequence that harbors the candidate causal mutation. *Red letters* between brackets represent the SNP at position 49,878,773 bp (*C*: reference allele, *T*: alternative allele)

On BTA4, *LRT* values exceeding the significance threshold of 25.223 were detected between SNPs at positions 46,172,128 and 54,324,740 bp, which indicated an association between this region and the occurrence of Weaver syndrome. The peak reached its maximum (*LRT* = 73.9) at position 49,812,384 bp and the boundaries of its confidence interval (CI) that were determined by the 2-LOD drop-off method were set at positions 49,514,652 and 50,367,484 bp (Fig. 1a). According to the *Ensembl* database [39], the following genes are located within this CI: *NRCAM*, *PNPLA8*, *NME8*, *SFRP4*, *EPDR1*, *STARD3NL*, the novel microRNA genes *ENSBTAG00000045095* and *ENSBTAG00000044255* and the uncharacterized protein coding genes *ENSBTAG00000014795* and *ENSBTAG00000035945* (Fig. 1b).

A second peak (*LRT* = 61.835) was detected outside of this first CI but in close proximity at position 47,598,264 bp. The boundaries of the CI of this secondary peak that were determined as above were set at positions 47,343,868 and 47,750,208 bp, which resulted in a 0.4-Mb CI that was found to harbor the following genes [39]: *CDHR3*, *SYPL1*, *NAMPT*, the microRNA gene ENSBTAG00000044548, the U6 snRNA gene *ENSBTAG00000042539* and the pseudogene *ENSBTAG00000019824*.

### Homozygosity mapping and identification of a common haplotype on BTA4

Homozygosity mapping using the complete set of Weaver cases from the *cLDLA* approach did not provide the expected results. It was necessary to exclude some animals which, upon re-examination, in fact were phenocopies, to successfully map the Weaver locus within a 1.913-Mb block of adjacent SNPs (between 48,408,626 and 50,412,884 bp) for which all remaining animals were homozygous (see Additional file 1: Figure S1). This interval was concordant with the common haplotype that was identified in parallel by manually scanning genotypes of affected animals (confirmed by all three diagnostic steps) for regions of homozygosity surrounding the main and secondary *LRT* peak on BTA4. By aligning the haplotypes of all progeny-tested Weaver carriers, the common haplotype was reduced to a region between SNPs at positions 48,688,283 and 50,412,884 bp. All Weaver-affected animals were homozygous at this 37-SNP haplotype, whereas animals designated as Weaver carriers carried only one copy of the haplotype. Furthermore, this haplotype analysis clearly excluded the above-mentioned secondary peak as a positional candidate region for Weaver syndrome (see Additional file 2: Figure S2).

### Exploiting whole-genome sequence data for the identification of candidate causal mutations

**Fig. 2:**
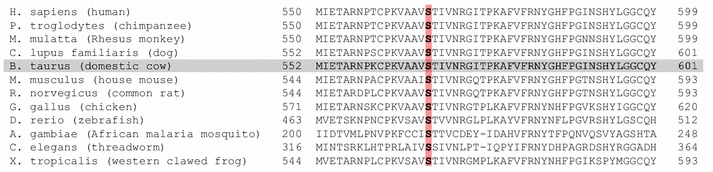
Multi-species alignment of the *PNPLA8* protein sequence. The *red bar* highlights the serine residue at position 568 of PNPLA8 which is conserved throughout eukaryotes. The *grey bar* highlights the *Bos taurus* reference sequence. (NCBI HomoloGene, http://www.ncbi.nlm.nih.gov/homologene, Accessed 25 Aug 2015)

We considered 22,160 sequence variants (21,151 SNPs and 1009 indels) that were located within the 1.72-Mb segment (between 48,688,283 and 50,412,884 bp) of extended homozygosity as positional candidate causal variants. Among these, only one variant, rs800397662 (Chr4:49,878,773 bp), was heterozygous in the two known Weaver carriers, while all other 1145 animals from 29 breeds were homozygous for the reference allele (see Additional file 3: Table S1). This only compatible variant is a missense mutation (p.S568N, c.G1703A) in the *PNPLA8* gene that encodes patatin-like phospholipase domain containing 8 (Fig. 1c, d). This p.S568N variant is predicted to be highly damaging to protein function (*SIFT*-score [40]: 0.01, *Polyphen2*-score [41]: 1.00). A serine residue at position 568 of *PNPLA8* is conserved throughout eukaryotes, which suggests that it is essential for normal protein function (Fig. 2).

Furthermore, a second variant (rs442854880) which was also exclusively heterozygous in the two known Weaver carriers and homozygous for the reference allele in all other sequenced animals was detected outside of, but in close proximity to, the region of extended homozygosity at position Chr4:50,858,538 bp.

### Targeted PCR-RFLP genotyping for SNP Chr4:49,878,773 bp (rs800397662)

The results of the PCR-RFLP assay agreed with the theory of a causative mutation at position 49,878,773 bp since carrier animals were heterozygous and affected animals were homozygous for the alternative allele, while Weaver-free animals were homozygous for the reference allele.

We observed conflicts for four Weaver-affected animals with pathological records and one Weaver carrier whose status had been determined only on the basis of the indirect microsatellite test (Table 1). Three of the affected animals were tested heterozygous (*CT*), while one affected animal and the carrier were tested homozygous (*CC*) for the reference allele of the SNP at position 49,878,773 bp.

**Table 1:** Conflicts between clinical/genetic diagnosis and targeted genotyping for the SNP Chr4:49,878,773 bp

Animal ID	Clinical or genetic diagnosis from the 1990s	Genotype at SNP_49878773	Genotype at SNP_50858538
W0045	Affected	*CT*	*GA*
W0068	Affected	*CT*	*GA*
W0084	Affected	*CC*	*GG*
W0125	Affected	*CT*	*AA*
W0226	Carrier (MS test)	*CC*	*GG*

Capital letters represent the animals’ genotypes (SNP_49878773 genotypes: *C*: reference allele, *T*: alternative allele; additional information on SNP_50858538 genotypes: *G*: reference allele, *A*: alternative allele). MS test: indirect genetic test for Weaver syndrome based on six microsatellite markers (RM188, MAF50, RM067, TGLA116, BM1224, BM6458); animals with an estimated risk of ≥95 % of carrying the Weaver allele were declared as carriers and excluded from further breeding

### Targeted PCR-RFLP genotyping for SNP Chr4:50,858,538 bp (rs442854880)

The results of the PCR-RFLP assay for the second candidate causal mutation at Chr4:50,858,538 bp, which is located outside the 1.72-Mb segment of extended homozygosity, showed that among the 30 progeny-confirmed Weaver carriers, all animals except one (W0037) were heterozygous at this SNP (*GA*) (Table 2). Animal W0037 is the dam of one pathologically-confirmed Weaver-affected animal although it is homozygous for the alternative allele *A*. Genotyping of the 16 Weaver-affected animals showed that they were all homozygous for the alternative allele (*AA*) except animal W0246 (Table 2) which was heterozygous (*GA*) at the SNP at Chr4:50,858,538 bp and for the region surrounding this SNP.

**Table 2:** Comparison between clinical/genetic diagnosis and targeted genotyping for the SNP Chr4:50,858,538 bp

Animal ID	Clinical or genetic diagnosis from the 1990s	Genotype at	
		SNP_49878773	SNP_50858538
W0037	Carrier	*CT*	*AA*
W0246	Affected	*TT*	*GA*

Capital letters represent the animals’ genotypes (additional information on SNP_49878773 genotypes: *C*: reference allele, *T*: alternative allele; SNP_50858538 genotypes: *G*: reference allele, *A*: alternative allele)

### Re-evaluation of pathological and pedigree records of animals with conflicting PCR-RFLP results

It should be noted that this study investigated tissues and histopathological sections that were collected and prepared over 20 years ago. For animals W0068, W0084 and W0125 (Table 1), neither histopathological sections nor embedded spinal cord tissue were stored in the archive of the LMU Institute of Veterinary Pathology. Records and pedigree data from a previous project on Weaver syndrome revealed that there were already serious doubts about the animals’ disease status at the time of collection. For these three animals, confirmed Weaver carriers were missing on one or both sides of the pedigree, which indicates that Weaver syndrome had been incorrectly diagnosed. We also assumed a false positive result for sire W0226 whose carrier status had been determined by the commercial microsatellite test although there were no reports of Weaver cases among its offspring [42].

For animal W0045, embedded spinal cord tissue was still available, thus newly prepared sections were evaluated by the Institute of Veterinary Pathology and confirmed the anticipated disease status as Weaver-affected. However, this did not agree with the results of the PCR-RFLP assay that showed that it was heterozygous (*CT*). Reassessment of familial relationships revealed that this animal was born as a twin, which raised the possibility of blood chimerism. Blood chimerism describes the coexistence of blood cell lines with two different genotypes in one organism [43] and is a phenomenon often observed in twin cattle [44]. DNA for animal W0045 was extracted from blood, which was the only available tissue, and was genotyped twice with Illumina’s BovineSNP50 BeadChip. Low call rates of 0.95 and 0.96, respectively, were obtained which is typical of chimeric individuals [45]. In order to genotype samples for the candidate SNP at position 49,878,773 bp (rs800397662) more efficiently, a specifically designed KASPar™ Genotyping System (LGC, Teddington, UK) was used. The KASP assay [46] is known for its high correlation (average *r* = 0.9796 ± 0.0094) between relative allele dosage and the difference in observed signal strength [47]. Comparison of the KASP signal intensities of 39 confirmed Weaver carriers and animal W0045 revealed significant differences. While signal intensities were relatively similar for both alleles (*C*: 4478.84 and *T*: 4654.28) for all Weaver carriers, the intensity of the reference allele for animal W0045 was significantly weaker (*C*: 2605.50) than that of the alternative allele (*T*: 6412.00).

For animals W0037 and W0246, for which controversial results were found in targeted genotyping for the SNP at position 50,858,538 bp (rs442854880), it was not possible to perform a histopathological reassessment because no spinal cord tissue was available. Pedigree records revealed that animal W0037, which was homozygous for the alternative allele, had given birth to a calf at the age of 38 months. Since the first symptoms of Weaver syndrome usually appear between 5 and 8 months of age [6], i.e. before the onset of sexual maturity, the alternative allele at 50,858,538 bp was excluded as a candidate causal mutation. This exclusion was further supported by the genotyping results for animal W0246 which was homozygous for the reference allele, while all available pedigree and pathological records confirmed its status as a Weaver-affected animal.

### Estimation of the frequency of SNP Chr4:49,878,773 bp in a random sample of 2334 current Braunvieh animals

A customized version of the BovineSNP50 genotyping array was developed to test genotypes for several relevant mutations in the German and Austrian cattle populations including the mutation at Chr4:49,878,773 bp (rs800397662). The frequency of the *T* allele at SNP Chr4:49,878,773 bp was equal to 0.26 % in 2334 Braunvieh animals born between 2013 and 2015 that had been genotyped with this custom array since January 2014 (Table 3). For the remaining 12,197 animals from different breeds, the *T* allele was not detected.

**Table 3:** Estimation of the frequency of the mutation in *PNPLA8* in current Braunvieh/Brown Swiss animals

Year	Number of genotyped bulls	Number of heterozygous animals	Allele frequency (%)
2013	122	2	0.82
2014	834	3	0.18
2015	1378	7	0.25

Number of genotyped bulls according to year of birth (November 2015)

### Targeted genotyping on a random sample of animals from other breeds that carry the Weaver haplotype

The genotyping results with the custom chip clearly demonstrated the absence of the only remaining and most likely candidate causal mutation for Weaver syndrome in 12,197 non-Braunvieh animals. To further demonstrate that this mutation is also absent in carriers of the Weaver haplotype from other breeds, we scanned haplotypes of several non-Braunvieh animals in the Bavarian and Swiss databases and found six animals that carried the Weaver haplotype and for which samples could be easily obtained for targeted genotyping, i.e. four Fleckvieh, one Swiss Simmental and one Holstein. All six animals were homozygous for the reference *C* allele at Chr4:49,878,773 bp, thus an association between the Weaver haplotype and the most promising candidate causal mutation across breeds was excluded.

### Re-assessing the carrier status of the controversial Weaver carrier W0277

Animal W0277 (Table 4), which was sired by one of the most prominent Weaver carrier bulls in Germany (W0380), was itself a successful AI sire with 11,398 daughters in milk production in Germany [48] and about the same number of sons including 14 proven sires [49]. In the 1990s, indirect genetic testing and risk analysis based on six microsatellite markers and the resulting haplotypes declared that animal W0277 was a Weaver carrier. This finding did not agree with the high frequency of the Weaver allele observed in the Braunvieh population [17, 18] and with the large number of unsuspicious W0277 offspring at that time. Consequently, this result was questioned by some breeders and breeding organizations. However, it is only after testing animal W0277 as a Weaver carrier that two direct offspring (W0165 and W0364) and one granddaughter (W0289) were identified as Weaver-affected animals on the basis of clinical symptoms and subsequent histopathological examination of relevant tissues.

**Table 4:** Familial relationships, genotypes and assumed disease status of animal W0277, its progeny and their dams

Animal ID	Relationship between animals (see Fig. 3)	Clinical/genetic diagnosis from the 1990s	Clinical re-evaluation in 2015	Genotype of SNP at	
				49,878,773 bp	50,858,538 bp
W0277	Sire of W0165 and W0364, maternal grandsire of W0289	Carrier	–	*CC*	*GG*
W0165	Direct offspring	Affected	Free	*CC*	*GA*
W0166	Dam of W0165	Carrier	–	*CC*	–
W0364	Direct offspring	Affected	Free	*CC*	*GG*
W0365	Dam of W0364	Carrier	–	*CC*	*GG*
W0289	Granddaughter of W0277	Affected	Free	*CC*	–
W0287	Dam of W0289, daughter of W0277	Carrier	–	*CC*	–
W0303	Sire of W0289	Carrier	–	*CT*	*GG*

SNP_49878773 genotypes: *C*: reference allele, *T*: alternative allele; SNP_50858538 genotypes: *G*: reference allele, *A*: alternative allele, dashes indicate that the animal was not genotyped due to the lack of testing material. Clinical re-evaluation in 2015: dashes indicate the lack of embedded spinal cord tissue and histopathological sections

These findings on the suspected carrier bull W0277 and its three offspring challenged our analyses. In order to clarify the carrier status of W0277 and eliminate any uncertainty with respect to the small number of affected offspring, samples from this bull, three of its progeny and their corresponding dams were also analyzed in our study. Hematoxylin–eosin-stained histological sections of the spinal cord at three different levels (lumbar, thoracic and cervical sections of the spinal cord) were re-evaluated by the LMU Institute of Veterinary Pathology in the light of the latest scientific knowledge. In a parallel approach, BovineSNP50 and PCR-RFLP genotypes were collected using available DNA samples of W0277 and its relatives (Table 4).

Neuropathological re-evaluation of histological sections showed atypically-located lesions in the dorsal part of the spinal cord or the grey matter, edematous alterations and inflammation. These findings pointed more towards spinal cord infarction or segmental damage through trauma rather than towards true cases of Weaver syndrome. In addition, no common type and distribution pattern of the lesions were identified.

**Fig. 3:**
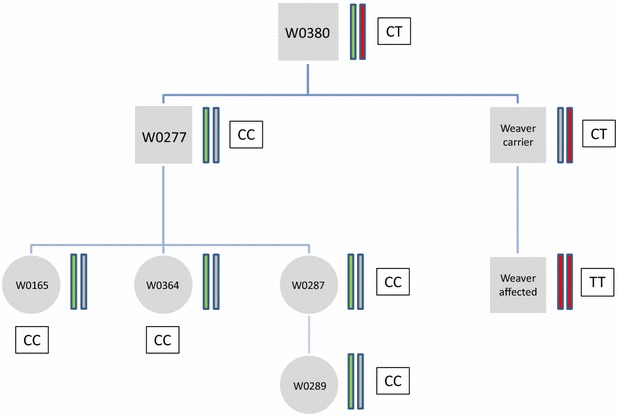
Familial relationships, genotypes and haplotype structure of animal W0277 and its progeny. Haplotypes in *red* represent the common Weaver haplotype identified by homozygosity mapping (48,688,283–50,412,884 bp), haplotypes in *green* represent the haplotype that was passed down from animal W0380 to W0277 and inherited by its progeny and haplotypes in grey represent all haplotypes that were inherited from non-carrier and non-affected ancestors. *Letters* in *rectangles* represent the animals’ genotypes for the SNP at position 49,878,773 bp (rs800397662) with *C* as the reference allele and *T* as the alternative allele

The results of the PCR-RFLP assay for SNP Chr4:49,878,773 bp showed that the affected progeny, their respective dams and the W0277 bull were homozygous for the reference allele, which did not match with their assumed disease status. Only the sire of the affected granddaughter was heterozygous, which agreed with its carrier status (Table 4). Moreover, the absence of the Weaver haplotype in the W0277 family confirmed these findings (Fig. 3).

Subsequent homozygosity mapping of the three W0277 offspring against a set of 46 healthy Brown Swiss and 34 Original Braunvieh animals revealed only three relatively short haplotypes (that contained five or six consecutive SNPs) for which all three W0277 offspring were exclusively homozygous. These haplotypes were located on BTA1 (between 139,231,307 and 139,409,122 bp), BTA5 (between 10,560,048 and 10,788,597 bp) and BTA16 (between 62,134,743 and 62,361,331 bp). Comparison of the DNA sequences of each of these chromosomal segments between animal W0277 and its Weaver carrier sire did not identify any known mutation that may cause a fatal recessive disorder based on current knowledge. Therefore, all three W0277 offspring represent phenocopies and are characterized by the absence of any common genetic or phenotypic feature.

## Discussion

Combined linkage/linkage disequilibrium mapping on BTA4 resulted in a log-likelihood peak at position 49,812,384 bp (Fig. 1a). The corresponding CI (between 49,514,652 and 50,367,484 bp) contained two genes that were previously discussed by McClure et al. [23] as two of the most likely candidate genes for Weaver syndrome: *NRCAM* and *PNPLA8*, with *PNPLA8* being more closely located to the position of the maximum *LRT* value (Fig. 1b). Although gene products of *NRCAM* play important roles in neuronal development and function [35, 50–54], this gene was excluded as a potential candidate following the results presented here.

Furthermore, a common homozygous haplotype that was shared by all Weaver cases was identified. The 1.72-Mb segment (between 48,688,283 and 50,412,884 bp), which overlapped with the diagnostic haplotype that had been suggested by McClure et al. [23] and the CI of the main peak along its entire length, excluded the secondary peak that was detected at position 47,598,264 bp (*LRT* = 61.835) (see Fig. 1a, Additional file 1: Figure S1). Of the two candidate mutations that were identified by analyzing whole-genome sequence data from the 1000 bull genomes project [38], the non-synonymous SNP at position 49,878,773 bp (rs800397662) in *PNPLA8* was included in the 1.72-Mb segment of extended homozygosity (Fig. 1b), while the second mutation (rs442854880) was located close to the haplotype’s distal border at position 50,858,538 bp in *CTTNBP2*. Both mutations were also on a list of SNPs located between 48 and 53 Mb on BTA4 which had been suggested as candidate causal variants by McClure et al. [23] in 2013. Considering the expression pattern and biological functions of the corresponding gene products, they had concluded that the SNP in *PNPLA8* was one of the most likely variants involved in Weaver syndrome [23]. Targeted SNP genotyping by PCR-RFLP revealed conflicting results for both mutations (Tables 1, 2). However, for the mutation at position 49,878,773 bp, the conflicts could be resolved by analyses of pathological records, pedigrees and genotypes that supported the hypothesis of phenocopies and by the detection of blood chimerism. Equivalent analyses for the SNP at 50,858,538 bp led to its exclusion as a candidate causal mutation.

Overall, the results of our analyses largely agree with the findings of McClure et al. [23] and are in favor of the non-synonymous SNP at 49,878,773 bp (rs800397662) as the most likely candidate causal mutation for Weaver syndrome. Similarities between the symptoms that are observed for this disease and the biological processes that are affected by knock-out of *PNPLA8* further support this theory. *PNPLA8* encodes calcium-independent phospholipase A_2γ_ (iPLA_2γ_), which is a member of the phospholipase A2 family. These enzymes catalyze the hydrolytic cleavage of membrane glycerophospholipids at the sn-2 position into free fatty acids and lysophospholipids which can be further converted to various biologically active molecules and signaling metabolites [55]. Activity of iPLA_2γ_ is linked to the release of arachidonic acid and the production of several eicosanoids and lysolipids involved in mitochondrial bioenergetics and signaling [56]. In vitro analyses of rabbit renal proximal tubular cells with suppressed expression of iPLA_2γ_ revealed increased lipid peroxidation and apoptosis, suggesting a critical role of the enzyme in the protection of cells against oxidative stress and the subsequent repair processes [51]. Similar results were observed in iPLA_2γ_^-/-^ mice that showed impaired mitochondrial function [53, 57] and increased levels of oxidative stress [57] leading to progressive loss of muscular function and a phenotype displaying bioenergetic dysfunction [53]. In humans, a case of compound heterozygosity for two frameshift mutations in *PNPLA8* was published recently [58]. The affected patient suffered from progressive muscle weakness, gait abnormalities and other neurodegenerative symptoms which were absent at birth but became apparent during the first years of infancy. Analyses revealed a lack of iPLA_2γ_ in the muscle, ultrastructural abnormalities in the mitochondria and a general resemblance with symptoms observed in the above-mentioned iPLA_2γ_^-/-^ mice, which suggests that there is a link between the patient’s phenotype and the mutations in *PNPLA8* [58].

Based on our assumption that the SNP in *PNPLA8* (rs800397662) is the causal mutation, the disease status of a controversial carrier bull and its progeny, that were diagnosed as Weaver-affected animals but were homozygous for the reference allele, was re-evaluated. The results of the histopathological examination of sections of the spinal cord of three of its progeny disagreed with the original diagnosis (Table 4), which suggested that they were in fact phenocopies which had been misdiagnosed under the pressure to identify and eliminate all possible Weaver carriers from breeding during the late 1980s and early 1990s. However, their high degree of relationship (Fig. 3) was both interesting and challenging and led us to put forward the following hypotheses: (1) all three phenocopies are the result of a different heritable disease which needs to be further clarified; or (2) all three phenocopies are induced by a non-genetic cause. Regardless of the scenario, animal W0277 was incorrectly assigned a carrier status. Since we did not identify any common histopathological pattern, i.e. we observed three patterns that all differed from the typical Weaver diagnosis and were better explained by infarctious damage and/or trauma to the spinal cord, the theory of another heritable disease that mimics Weaver syndrome was discarded. In addition, homozygosity mapping against a control population of healthy individuals and comparison of the intervals identified with whole-genome sequence data from animal W0277 did not lead to any obvious candidate variants, which supports the conclusion that a second mutation or an unknown genetic disease in the investigated family of W0277 is rather unlikely.

Our study greatly benefited from the availability of preserved spinal cord tissue, pathological records and a large number of blood and DNA samples, especially from animals affected by Weaver syndrome. As a result, we were able to detect the above-mentioned phenocopies which had been misdiagnosed in the 1990s and which may also explain why none of our earlier mapping attempts were successful. It is only through the combination of exhaustive sequencing data, linkage/linkage disequilibrium mapping and histopathological examination that we were able to provide supporting evidence that the non-synonymous mutation at position 49,878,773 bp (rs800397662) in *PNPLA8* is the only remaining and most likely candidate causal mutation for Weaver syndrome. Our results further demonstrate several advantages of the *cLDLA* approach over conventional homozygosity mapping, with the most important being that *cLDLA* can exploit information that is contributed by confirmed carriers and tolerate the presence of phenocopies in the mapping design. Moreover, there is a significant difference in mapping resolution between both methods, i.e. *cLDLA* is more precise even after excluding the phenocopies from the homozygosity mapping design (see Additional file 1: Figure S1).

Monitoring of the presumed causal mutation within a sample of current Braunvieh/Brown Swiss candidates for genomic selection shows that in spite of its low frequency in the genetically active part of the population (0.26 %; Table 3), it is still present. Among the 12 new Weaver carriers that were detected with the customized chip, two could have been selected as AI bulls based on their high genomic breeding values if they had not been tested for Weaver syndrome. Consequently, although the probability that two carriers mate and produce affected offspring is much lower (approximately one out of ~605,000) than previously anticipated [22], this mutation still remains an important monitoring criterion of the population using the custom chip. It can also be assumed that over the last two decades, not all affected animals were detected and reported. Speaking from our general experience, reporting discipline strongly depends on the breeders’ current level of education and sensitization on a certain disease, which, in the case of the Weaver syndrome, is probably very low since many breeders and veterinarians may currently not be able to recognize a case of Weaver syndrome.

Although the common haplotype was detected in other breeds, such as the Fleckvieh and Holstein breeds, at a very low frequency (0.004 in Fleckvieh and 0.001 in Holstein), targeted genotyping showed that the candidate causal mutation is not present in these breeds. Consequently, scanning populations for the common haplotype is not sufficient for Weaver diagnosis and needs to be complemented by other analyses. According to the cumulative evidence of previous research, this might be a general rule that is also valid for other monogenic phenotypes that segregate in small domestic breeds.

From a breeding point of view, most of the questions regarding this recessive disorder have found answers with the findings of our study and previous ones. Nevertheless, basic issues transcending the species and regarding an in-depth understanding of the cellular and molecular genetic mechanisms involved in this progressive neurodegenerative disease still remain. To fully explain the link between the altered *PNPLA8* gene product and the symptoms observed in Weaver-affected animals and thus further confirm the causal character of this mutation, additional research on the cellular mechanisms that are involved is necessary. For this purpose, there are several possibilities including state-of-the-art analyses of old paraffin-embedded tissue samples, in vitro gene editing and, if close monitoring of risk matings that involve the carriers detected here revealed any current Weaver cases, examination of live Weaver-affected animals.

## Conclusions

Based on the results of both McClure et al. [23] and our studies, the non-synonymous SNP in *PNPLA8* (rs800397662, 49,878,773 bp) is most likely the causative mutation that underlies Weaver syndrome. The perfect association between this SNP that was detected by whole-genome sequencing, different mapping and genotyping approaches and the cellular functions of the *PNPLA8* gene products highly supports this conclusion. Estimation of allele frequency in a sample of current Braunvieh/Brown Swiss animals for the time span between 2013 and 2015 resulted in a low value of 0.26 %. With a very low expected risk of random carrier–carrier mating (0.0000066), the occurrence of new Weaver cases after more than 20 years of complete absence of case reports seems rather unlikely but corresponds to approximately one case per 605,000 animals, i.e. at most one Weaver case per year in the current German-Austrian Braunvieh cow population. The presence of the Weaver haplotype alone is not sufficient for an indirect diagnosis neither in the Braunvieh/Brown Swiss breed nor in any other cattle breed. Future research on the cellular mechanisms that are altered by this non-synonymous mutation in *PNPLA8* is needed to further prove its causal character and thus enhance our understanding of the cellular functions that are involved in Weaver syndrome and similar neurodegenerative diseases in various species including humans.

## Additional files

10.1186/s12711-016-0201-5 Comparison of the results of *cLDLA* mapping with the results of case–control homozygosity mapping. The blue line with blue dots represents the results of the *cLDLA* mapping approach using data on 43 Weaver-affected, 31 Weaver carriers and 86 Weaver-free animals which resulted in a maximum *LRT* value (*LRT* = 73.9) at position 49,812,384 bp. The corresponding 0.853-Mb confidence interval between SNPs at 49,514,652 and 50,367,484 bp, represented by the grey bar, is assumed to harbor the causative mutation. The black line with black squares represents the results of a case–control homozygosity mapping approach [37] based on all Weaver-affected (case group) and Weaver-free animals (control group) from the *cLDLA* mapping population. In a second run, all Weaver cases which had been identified as phenocopies in the course of our further analyses were removed from the case group. The results of this run are displayed by the red line with red squares. Only run 2 of the homozygosity mapping approach, i.e. after removal of the phenocopies which were identified later, displayed a significant association with a 1.913-Mb block of adjacent SNPs (between 48,408,626 and 50,412,884 bp). However, the *cLDLA* approach was able to map the Weaver locus to the same chromosomal region even if the phenocopies were still included in the mapping population. Furthermore, the 0.853-Mb confidence interval identified by the *cLDLA* method allows for more precise analyses than the 1.913-Mb block of SNPs identified by homozygosity mapping, accounting for the higher mapping resolution obtained by the *cLDLA* method.
10.1186/s12711-016-0201-5 Identification of a common haplotype on BTA4. Description: Genotypes of a subset of 32 Weaver carriers and 13 Weaver-affected offspring were scanned for regions of homozygosity surrounding the main and secondary peaks. The segment of interest consisted of 197 SNPs between positions 44,387,787 and 47,598,264 bp. Carets (^) indicate the position of the secondary peak (47,598,264 bp), asterisks (*) indicate the position of the main peak (49,812,384 bp). Red bars indicate shared haplotype segments, green bars indicate the common haplotype shared by both Weaver-affected and carrier animals.
10.1186/s12711-016-0201-5 Genotype distribution of 41 candidate causal mutations for Weaver syndrome in 1147 animals. The distribution of the genotypes of 41 candidate causal mutations (McClure et al. [23]) for Weaver syndrome in 1147 animals sequenced in the course of the 1000 bull genomes project was analyzed. The sequenced animals were grouped by breed. Numbers represent the within-breed frequency of the alternative allele. The distribution of genotypes for each breed is given in parentheses (homozygous animals for the reference allele | heterozygous animals | homozygous animals for the alternative allele). The grey background indicates candidate causal variations within the 1.72-Mb segment of extended homozygosity. Blue color indicates two variants that were perfectly associated with the Weaver status of 1147 sequenced animals. However, only one compatible variant (rs800397662, Chr4:49,878,773 bp) is located within the segment of extended homozygosity.
