# Supplementary figures and images for: Confirmation of a non-synonymous SNP in PNPLA8 as a candidate causal mutation for Weaver syndrome in Brown Swiss cattle
Source: Genet Sel Evol. 2016 Mar 18;48:21. doi: 10.1186/s12711-016-0201-5 (PMC4797220; doi:10.1186/s12711-016-0201-5)

## Slide 1
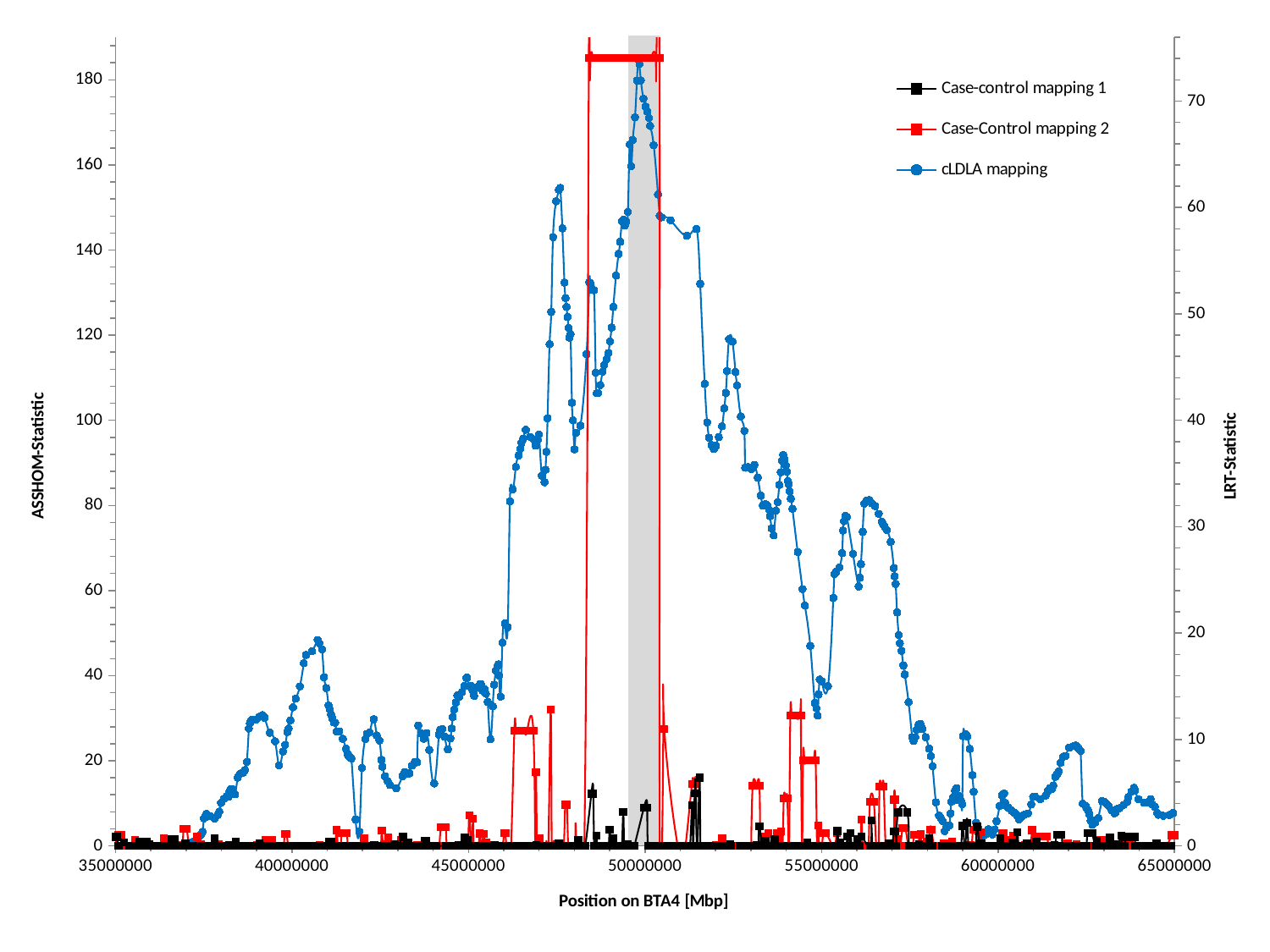

### Chart
| Category | Case-control mapping 1 | Case-Control mapping 2 | cLDLA mapping |
|---|---|---|---|

Supplement: Supplementary file 1 — 10.1186/s12711-016-0201-5 Comparison of the results of cLDLA mapping with the results of case–control homozygosity mapping. The blue line with blue dots represents the results of the cLDLA mapping approach using data on 43 Weaver-affected, 31 Weaver carriers and 86 Weaver-free animals which resulted in a maximum LRT value (LRT = 73.9) at position 49,812,384 bp. The corresponding 0.853-Mb confidence interval between SNPs at 49,514,652 and 50,367,484 bp, represented by the grey bar, is assumed to harbor the causative mutation. The black line with black squares represents the results of a case–control homozygosity mapping approach [37] based on all Weaver-affected (case group) and Weaver-free animals (control group) from the cLDLA mapping population. In a second run, all Weaver cases which had been identified as phenocopies in the course of our further analyses were removed from the case group. The results of this run are displayed by the red line with red squares. Only run 2 of the homozygosity mapping approach, i.e. after removal of the phenocopies which were identified later, displayed a significant association with a 1.913-Mb block of adjacent SNPs (between 48,408,626 and 50,412,884 bp). However, the cLDLA approach was able to map the Weaver locus to the same chromosomal region even if the phenocopies were still included in the mapping population. Furthermore, the 0.853-Mb confidence interval identified by the cLDLA method allows for more precise analyses than the 1.913-Mb block of SNPs identified by homozygosity mapping, accounting for the higher mapping resolution obtained by the cLDLA method. [file 12711_2016_201_MOESM1_ESM.pptx]

## Slide 1
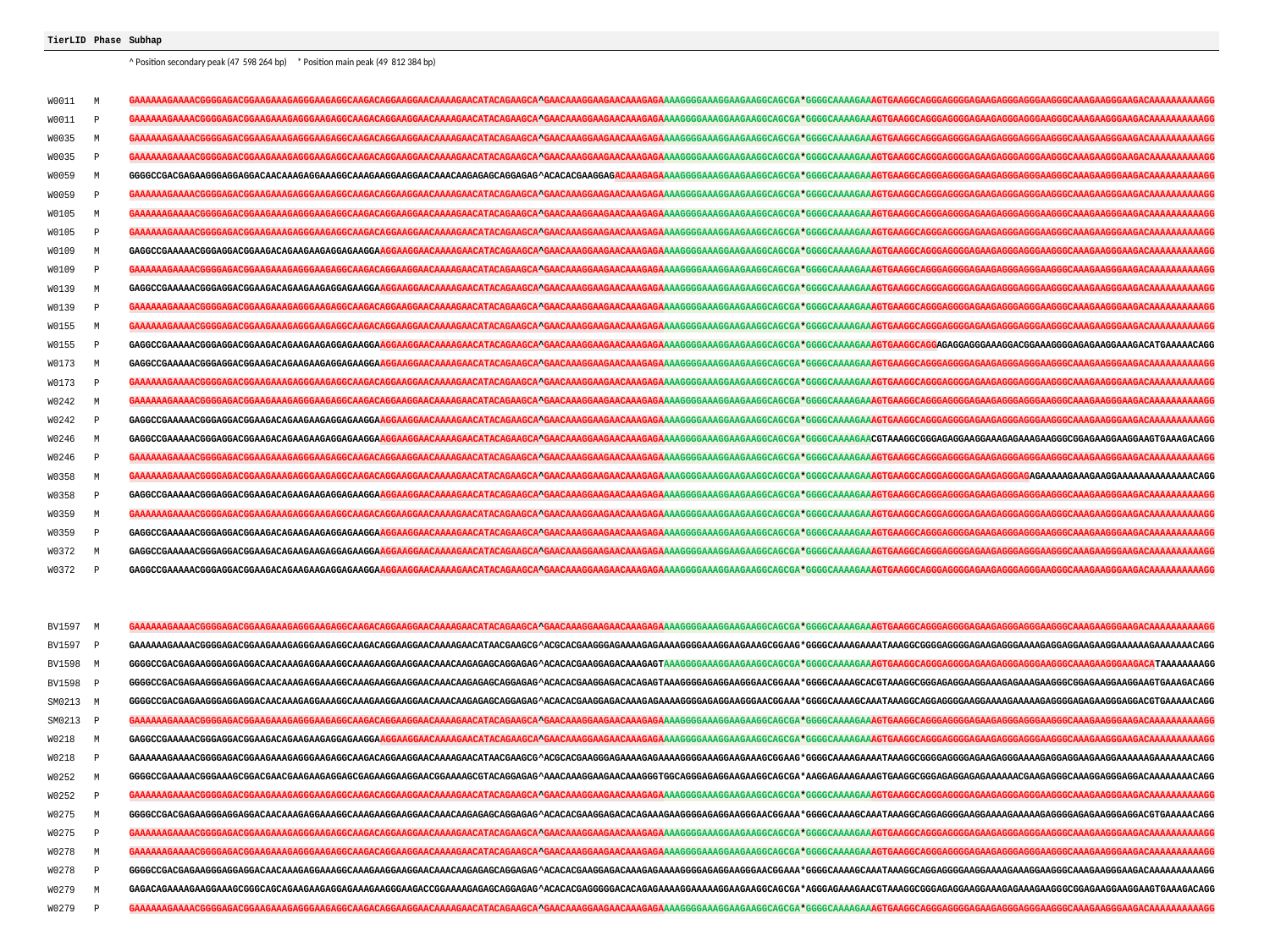

## Slide 2
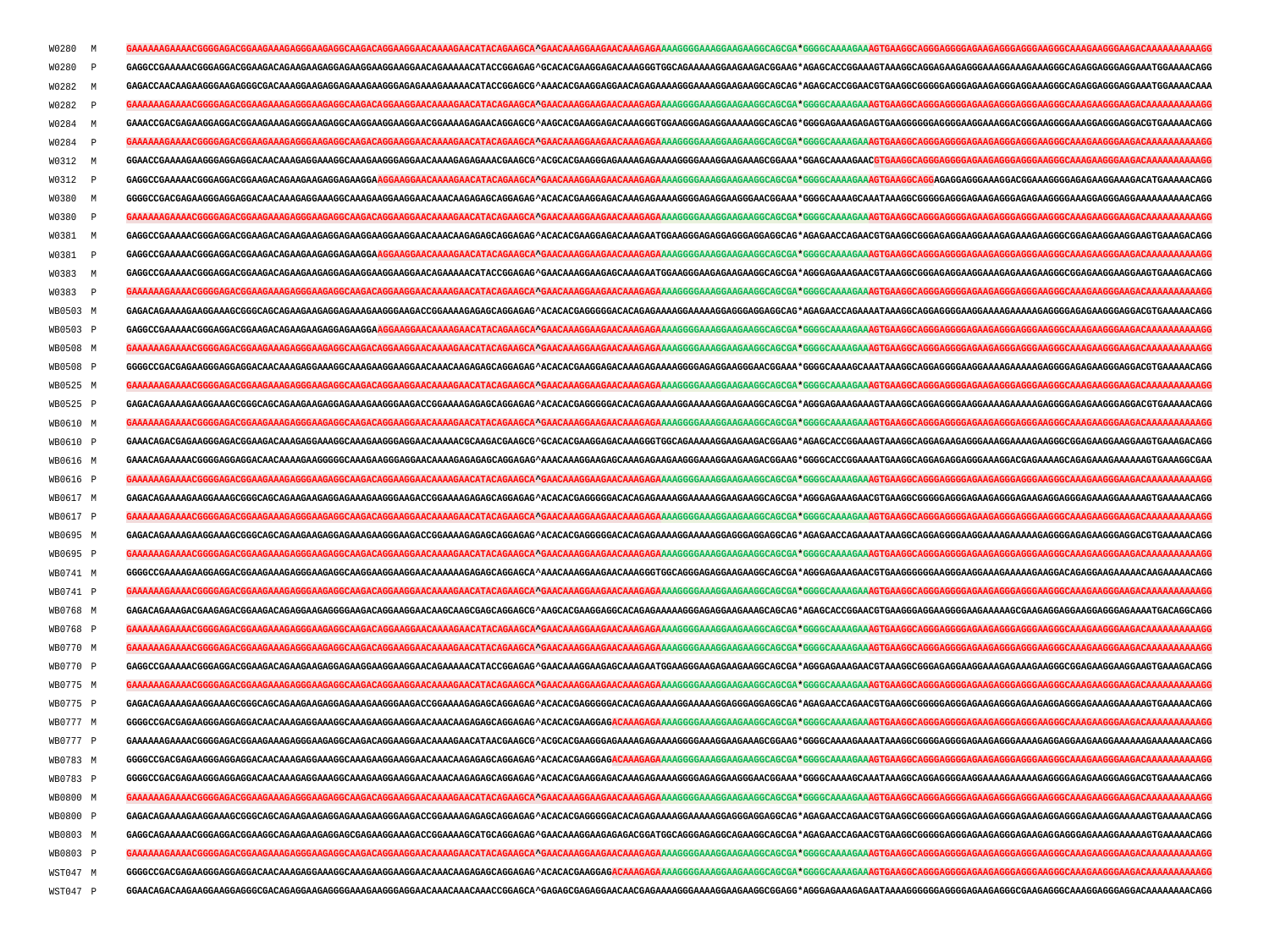

Supplement: Supplementary file 2 — 10.1186/s12711-016-0201-5 Identification of a common haplotype on BTA4. Description: Genotypes of a subset of 32 Weaver carriers and 13 Weaver-affected offspring were scanned for regions of homozygosity surrounding the main and secondary peaks. The segment of interest consisted of 197 SNPs between positions 44,387,787 and 47,598,264 bp. Carets (^) indicate the position of the secondary peak (47,598,264 bp), asterisks (*) indicate the position of the main peak (49,812,384 bp). Red bars indicate shared haplotype segments, green bars indicate the common haplotype shared by both Weaver-affected and carrier animals. [file 12711_2016_201_MOESM2_ESM.pptx]
